# Targeting Thyroid Hormone/Thyroid Hormone Receptor Axis: An Attractive Therapy Strategy in Liver Diseases

**DOI:** 10.3389/fphar.2022.871100

**Published:** 2022-06-02

**Authors:** Qianyu Tang, Min Zeng, Linxi Chen, Nian Fu

**Affiliations:** ^1^ Department of Gastroenterology, The Affiliated Nanhua Hospital, Hunan Provincial Clinical Research Center of Metabolic Associated Fatty Liver Disease, Hengyang Medical School, University of South China, Hengyang, China; ^2^ Department of Gastroenterology, Liuyang Hospital of Chinese Medicine, Changsha, China; ^3^ Department of Pharmacy and Pharmacology, Hunan Provincial Key Laboratory of Tumor Microenvironment Responsive Drug Research, Hunan Province Cooperative Innovation Center for Molecular Target New Drug Study, School of Basic Medical Science, Hengyang Medical School, University of South China, Hengyang, China; ^4^ The Affiliated Nanhua Hospital, Laboratory of Liver Disease, Institute of Clinical Research, Hengyang Medical School, University of South China, Hengyang, China

**Keywords:** TH/TR axis, thyroid hormone receptor, thyroid hormone, metabolic-associated fatty liver disease, hepatocellular carcinoma

## Abstract

Thyroid hormone/thyroid hormone receptor (TH/TR) axis is characterized by TH with the assistance of plasma membrane transporters to combine with TR and mediate biological activities. Growing evidence suggests that TH/TR participates in plenty of hepatic metabolism. Thus, this review focuses on the role of the TH/TR axis in the liver diseases. To be specific, the TH/TR axis may improve metabolic-associated fatty liver disease, hepatitis, liver fibrosis, and liver injury while exacerbating the progression of acute liver failure and alcoholic liver disease. Also, the TH/TR axis has paradoxical roles in hepatocellular carcinoma. The TH/TR axis may be a prospecting target to cure hepatic diseases.

## Introduction

Thyroid hormones (THs), including thyroid hormones 3,5,3′,5′- tetraiodothyronine or thyroxine (T4) and 3,5,3′-triiodothyronine (T3), are secreted by the thyroid gland to mediate homeostasis of biological growth, development, and metabolism (Senese et al., 2019; Turan and Turksoy, 2021). Thyroid hormone receptor (TR), a member of the nuclear receptor superfamily, is a ligand-dependent transcriptional factor. TR isoforms include TRα1, TRα2, TRβ1, TRβ2, and v-erbA (Ventura-Holman et al., 2011; Knabl et al., 2020). TRα and TRβ are encoded by chromosome 17 and chromosome 3, respectively (Onigata and Szinnai, 2014). V-erbA, acting like a transcriptional suppressor, is a derivant after TRα1 is affected by the avian erythroblastosis virus (AEV) (Ciana et al., 1998). TRα1 is mainly expressed in most peripheral organs except the liver, while TRβ1 is highly expressed in the liver. Although the TRα1 and TRβ1 mRNA levels are similar in metabolically active fats and muscles, protein levels are quite different (TRβ: TRα = 1:10). Moreover, TRβ2 is highly expressed in the pituitary gland, and gender differences in the expression have been found (Minakhina et al., 2020). The active form of TH, T3, and its nuclear receptor assembles ligand-dependent TH/TR complexes, thus regulating gene expression and directing downstream transcriptional activities (Lin et al., 2020a). In addition, thyroid hormone-response element (TRE) is located on the promoters of T3 target genes and affects the activity of the TR transcription response (Cheng, 2005). In pituitary gland, thyroid-stimulating hormone (TSH) is responsible for synthesis and secretion of TH while the encoding genes of TSH are also regulated by TH in a negative way (Bargi-Souza et al., 2017).

Dysfunction of the TH/TR axis leads to numerous pathologies, especially including growth, skeletal development, heart diseases, cognitive dissonance, gastrointestinal function, obesity, dysmetabolism, and cancers (Brent, 2012; Ortiga-Carvalho et al., 2014; Ajdukovic et al., 2021; Moutzouri et al., 2021; Niedowicz et al., 2021; Salman et al., 2021). Therefore, the abnormity of the TH/TR axis elicits a series of diseases, the most common of which is metabolic disease (Malm, 2004). The correlation between the TH/TR axis and many metabolism-associated diseases has been well-elucidated. For example, the TH/TR axis plays a protective role in hyperlipidemia, obesity, and type 2 diabetes (Grover et al., 2007). Intriguingly, the TH/TR axis is intimately associated with the development of the brain and the cerebellar both in fetal and adults (Ishii et al., 2021). Also, the TH/TR axis acts as a promoter in arrhythmia, gastric tumors, and alcoholic-related liver injury (Puhr et al., 2020; Deng et al., 2021). Considering the aforementioned facts, the TH/TR axis may be an indispensable part in maintaining hepatic metabolism.

Indeed, accumulating evidence has demonstrated that the TH/TR axis plays an important role in liver diseases. For instance, TRβ1, a subtype of TH, is highly expressed in the liver, regulating the metabolism of cholesterol and carbohydrates (Dawson and Parini, 2018; Gautherot et al., 2018). Additionally, the TH/TR axis, a strong inducer of hepatic autophagy contributing to lipid droplet degradation, as well as maintaining mitochondrial biogenesis and turnover, causes the removal of damaged mitochondria and ROS, ultimately preventing hepatic injury (Chi et al., 2019). As the TH/TR axis is correlated with various hepatic physiological alterations, more emphasis should be placed on the mechanism of the TH/TR axis in liver diseases. This review summarizes the regulatory mechanism of the TH/TR axis in the liver and focuses on the role of the TH/TR axis in hepatic diseases.

## TH/TR Axis Promotes Hepatocyte Proliferation and Liver Regeneration

**FIGURE 1:**
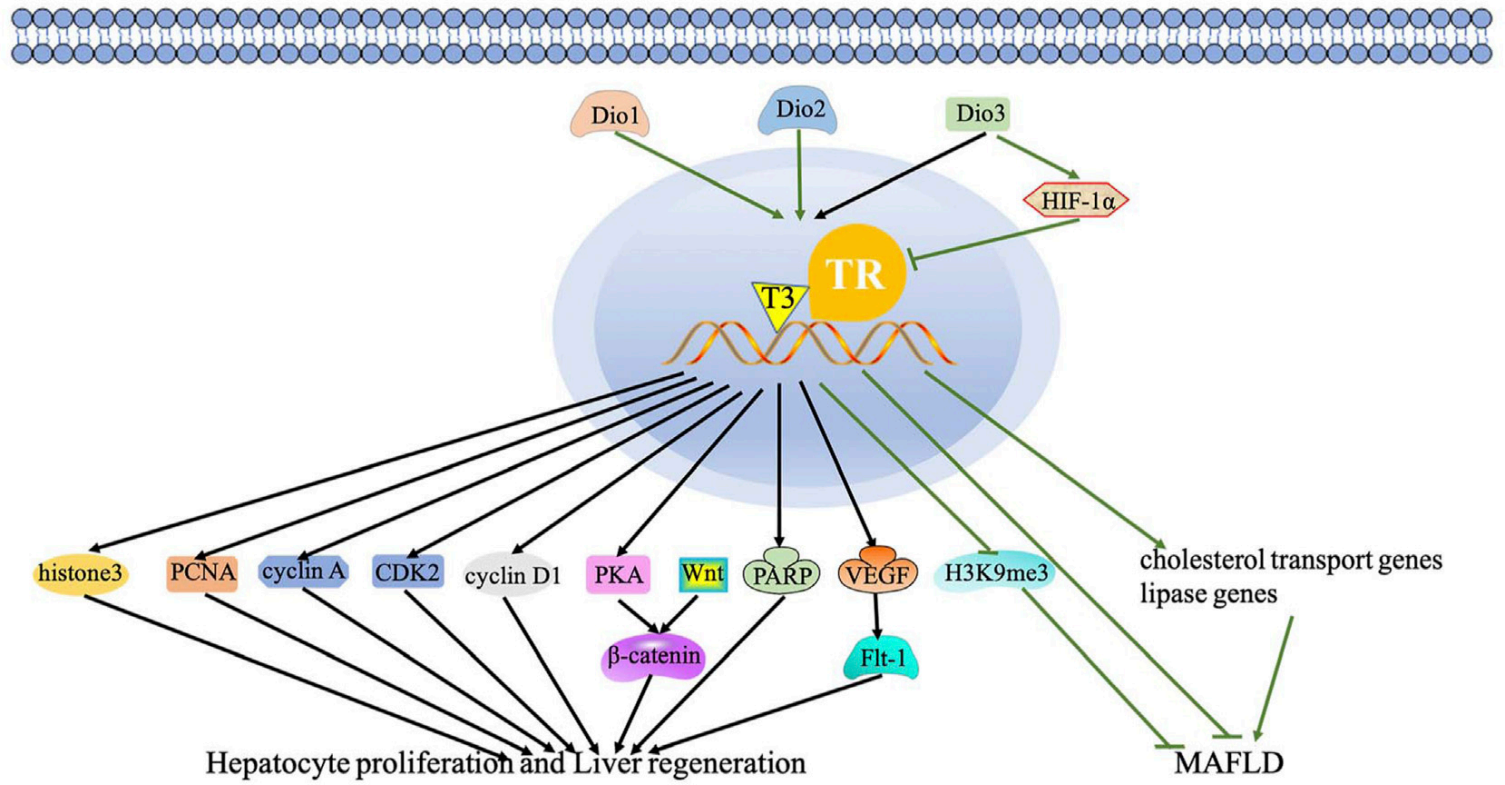
Regulatory mechanism of the TH/TR axis in hepatic proliferation, liver regeneration, and MAFLD. The black lines depict the following: the effect of the TH/TR axis in hepatic proliferation and liver regeneration. TH promotes liver regeneration *via* elevating histone 3 mRNA, PCNA, cdk2, cyclin A, and cyclin D1. The axis activates β-catenin to induce hepatocyte proliferation through PKA and Wnt-dependent mechanisms. PARP participates in liver regeneration induced by TH. The decrease of Dio3 elicits TH-dependent hepatocyte proliferation and liver regeneration. T3 improves liver regeneration by promoting the VEGF and Flt-1 expression. The green lines represent the following: the TH/TR axis might improve MAFLD. Dio3 activates HIF-1a, thus inhibiting T3 signaling. Genes related to reverse cholesterol transport and lipase activity decrease with the downregulation of Dio2 in rats. TH is produced by Dio2 and then depletes H3K9me3. (For interpretation of the references to color in this figure legend, the reader is referred to the Web version of this article).

TH has proven to be a hepatic mitogen, thus eliciting hepatocyte proliferating and liver repopulation. López-Fontal et al. (2010) also discovered that hypothyroidism and TR-deficient mice showed delayed recovery of liver mass. Interestingly, hypothyroidism can induce moderate non-alcoholic steatohepatitis, thereby promoting liver regeneration (Rodríguez-Castelán et al., 2017). A large number of studies reported that TRβ is involved in liver regeneration by the TH/TR axis (Sun et al., 2007). For instance, two TRβ agonists, TG68 and IS25, promote hepatocyte proliferation without TH/TR axis-dependent side effects (Perra et al., 2020). This aforementioned finding hints that the regulation of hepatocyte proliferation by the TH/TR axis is of great importance. Accordingly, studies have suggested the effect of the TH/TR axis on hepatocyte proliferation that TH promotes liver regeneration after 50% liver transplantation in mice *via* elevating histone 3 mRNA, proliferating cell nuclear antigen (PCNA), cyclin-dependent kinase 2 (cdk2), cyclin A, and cyclin D1 levels (Oren et al., 1999; Columbano et al., 2008; Taki-Eldin et al., 2011). The TH/TR axis activates β-catenin to induce hepatocyte proliferation through PKA and Wnt-dependent pathways (Fanti et al., 2014; Alvarado et al., 2016). Moreover, poly (ADP-ribose) polymerase (PARP), a nuclear enzyme involved in cell replication, is involved in the early steps of liver regeneration induced by TH after partial hepatectomy (PH) (Cesarone et al., 2000). The decrease of Dio3 elicits TH-dependent hepatocyte proliferation and liver regeneration (Kester et al., 2009). In addition, T3 bounds to nucleoprotein and then changes the interaction between nucleoprotein and TRE during liver regeneration (Hirose-Kumagai et al., 1995). These studies show that the regulation of hepatocyte proliferation by the TH/TR axis has been gradually demonstrated. Otherwise, the TH/TR axis is also involved in some specific regulation mechanisms of liver regeneration. For instance, Anan et al. (Abu Rmilah et al., 2020) summarized that TH mediates cell cycle regulators and apoptosis in liver regeneration. In addition, T3 improves liver regeneration by promoting the expression of VEGF and its receptor Flt-1 (Bockhorn et al., 2007). These studies suggest that the TH/TR axis may protect hepatocyte proliferation and liver regeneration (Figure 1).

## Interplay Between TH/TR Axis and Liver Diseases

Hepatocyte proliferation, regeneration, and lipid homeostasis in the liver are involved in many hepatic diseases. Significantly, numerous studies have shown that the prevalence and development of hepatic diseases are related to TH/TR axis abnormity (Mishkin et al., 1981). The interplay between the TH/TR axis and liver diseases are summarized. These diseases mainly include metabolic-associated fatty liver disease (MAFLD), hepatocellular carcinoma (HCC), hepatitis of hepatitis B virus (HBV) and hepatitis C virus (HCV) infection, acute liver failure (ALF), liver fibrosis, alcoholic liver disease, and liver injury.

### TH/TR Axis Might Improve MAFLD

MAFLD, formerly named as non-alcoholic fatty liver disease, is a serious liver issue worldwide and will be the leading cause of liver transplantation in the forthcoming decades (Méndez-Sánchez and Díaz-Orozco, 2021). A large number of studies reported that TH regulates hepatic triglyceride and cholesterol metabolism (Zhao et al., 2020). Accordingly, TH increases the activity of hepatic lipase, thus enhancing lipid mobilization from fat droplets. Moreover, TR activation triggers free fatty acid transporting into the hepatocytes (Tanase et al., 2020). Considering the aforementioned facts, the TH/TR axis is likely to be intimately correlated with hepatic diseases such as MAFLD. Accumulating evidence has demonstrated that the TH/TR axis is involved in MAFLD. To be specific, MAFLD is positively related with hypothyroidism, elevated TSH, T3, and thyroid peroxidase antibody (TPOAb), and suppressed T4 (Gor et al., 2021; D'Ambrosio et al., 2021).

The fact that whether the TH/TR axis can be a risk factor in MAFLD is not clear. Martínez-Escudé A et al. (2021) reported that TSH, regarded as a risk factor of MAFLD, is involved in obesity, atherogenic dyslipidemia, metabolic syndrome (MetS), hypertransaminasemia, and altered cholesterol and triglycerides levels. Then, a recent research hint that TSH is a MAFLD risk factor but excludes the FT3 and FT4 levels (Tan et al., 2021). Intriguingly, the result in a middle-aged and elderly euthyroid subjects showed that high-normal FT3 and low-normal TSH independently predict the high incidence of MAFLD (Gu et al., 2021). In addition, Chao et al. thought that FT3 and FT4 are independent risk factors to MAFLD. Conversely, although the level of TSH in non-MAFLD and MAFLD subjects who are undergoing health examinations are significantly different, TSH is excluded as an independent risk factor of MAFLD (Zhang X. et al., 2020; Chao and Chen, 2021). As described earlier, there is still controversy to identify TSH and TH as independent risk factors of MAFLD.

Importantly, the TH/TR axis regulate hepatic lipid metabolism such as mitochondrial fatty acid β-oxidation, lipid autophagy, and expression of lipid-related genes (Sinha et al., 2012; Kagawa et al., 2018). Thus, selective TRβ agonists may improve hepatic lipid disorders and MAFLD (Kowalik et al., 2018; Senese et al., 2019; Ritter et al., 2020). These agonists include MGL-3196 (Taub et al., 2013; Raza et al., 2021; Kannt et al., 2021), MB07811 (Erion et al., 2007), KB-141 (Erion et al., 2007), sobetisome (GC-1) (Huang Y. Y. et al., 2013; Ferrara et al., 2018; Saponaro et al., 2020), KB2115 (Eprotirome) (Ladenson et al., 2010a; Senese et al., 2019), and DITPA (Ladenson et al., 2010b; Senese et al., 2019) (Table 1). The side effects of selective TRβ agonists mostly result from TRα-induced dose-dependent cardiac effects, muscle metabolism, and bone turnover (Erion et al., 2007; Kelly et al., 2014). In short, the TH/TR axis may act as a promising treatment method for MAFLD.

**TABLE 1:** Comparison of different effects on selective TRβ receptor agonists.

Drug category (or categories)	Type of agonist/affinity	Effects on			Supplement	Reference
		Heart	Thyroid hormone axis (THA)	Lipid metabolism		
MGL- 3,196 (Resmetirom)	TRβ -selective agonist (28- fold over TRα)	Non-cardiac electrocardiogram change	At the highest dose, reversible free T4 was reduced by 20%. No significant change in TSH, free T3, and thyroid axis dysfunction	LDL-cholesterol, non-HDL-cholesterol, apolipoprotein B, and triglycerides were reduced. Liver weight, hepatic steatosis, plasma alanine aminotransferase activity, and blood glucose were reduced. The dose of 80 mg has the greatest effect on lipid metabolism.	No effect on body weight. No dose- related adverse events, no changes in liver enzymes, and vital signs. Phase 2–3 clinical trials are under way. Effects on insulin resistance and dog cartilage abnormality are dispute.	Sinha et al. (2012); Kagawa et al. (2018); Ritter et al. (2020)
MB07811 (vk2809; precursor of KB-141)	TRβ agonists	No significant change	Total and free T4 levels were decreased by day 7, with both doses of MB07811 and remaining constant over the subsequent 6 weeks of treatment. Levels of TSH and TSH mRNA were reduced.	Decreased serum TGs, liver TGs, and liver weight	No effect on body weight, fasting blood glucose, plasma insulin and plasma FFA, SREBP-2, and HMG- CoA reductase or phosphoenolpyruvate carboxykinase in the liver	Kowalik et al. (2018)
KB-141	TRβ agonists	Increased heart rate, the first derivative of left ventricular pressure, and systolic aortic pressure, followed by reduced weight	Decreased total 3,5,3, 5-tetraiodo- l-thyronine (T4) and free T4, total T3, and free T3	Not liver TGs but lower serum TGs and liver weight	No difference in the maximum cholesterol lowering effect between KB-141 and MB07811.	Kowalik et al. (2018)
Sobetirome (GC- 1)	GC-1 binds TRβ higher than that of TRα	No undesirable effects	TRH surpression:T3>Sob -AM2>sobetirome, decreased or depleted circulating T4 and T3 levels without altered serum TSH levels	Reduced serum cholesterol triglyceride and lipoprotein (a) levels. Reverse very high-fat diet (VHFD)-induced fat accumulation in the liver and induced weight loss. Reverse cholesterol transport pathway	Hyperglycemia and insulin resistance. The drug was stopped after the first phase of clinical trial.	Taub et al. (2013), Fanti et al. (2014), Raza et al. (2021)
DITPA	Similar affinity to both TR isoforms with relatively low affinity	Increased cardiac index and decreased systemic vascular resistance	Lowered serum TSH levels, to a lesser extent, serum T3 and T4, and no differences in clinical manifestations of thyrotoxicosis or hypothyroidism	Decreased serum cholesterol, low-density lipoprotein cholesterol and body weight, and a transient decrease in triglycerides and no change in high-density lipoprotein cholesterol	Reduced body weight and dangerous skeletal actions	Erion et al. (2007), Senese et al. (2019)
KB2115 (Eprotirome)	KB2115 has modestly higher affinity for TRβ than for TRα	No undesirable effects	No adverse extrahepatic thyromimetic effects	Reduced serum total and LDL-cholesterol, apolipoprotein B, triglycerides, and Lp (a) lipoprotein, prevents hepatic steatosis	Increase in transaminase and conjugated bilirubin concentrations; clinical trials were discontinued because long-term studies in dogs resulted in cartilage damage.	Senese et al. (2019), Kannt et al. (2021)

### TH/TR Axis Is Involved in HCC Growth, Proliferation, Invasion, and Metastasis

HCC is one of the most common malignant tumors. The TH/TR axis is involved in HCC. Some studies have demonstrated that mutations of TR genes are associated with human carcinoma (Anyetei-Anum et al., 2018; Piqué et al., 2020). A clinical study exhibited that hypothyroidism delays hepatocyte growth while hyperthyroidism promotes HCC (Mishkin et al., 1981). Additionally, the level of TR expression in adenomas (83%) and cancer (68%) is significantly lower than that in normal epithelium (96%) (Liu et al., 2019). Moreover, TH-related mitochondrial turnover protects hepatocytes from HBV hepatocarcinogenesis (Chi et al., 2017; Hossain et al., 2020). In addition, the TH/TR axis regulates proliferation, differentiation, metastasis, and drug resistance, autophagy in HCC (Jazdzewski et al., 2011; Rosen and Privalsky, 2011; Jerzak et al., 2015; Liu et al., 2019; Lin et al., 2020a).

### TH/TR Axis May Be Involved in Hepatitis of Hepatitis B Virus and Hepatitis C Virus Infection

Hepatitis of HBV and HCV infection are global issues, which have a risk to develop severe liver disease such as liver cirrhosis and HCC (Jing et al., 2020; Zhang et al., 2021). A study hinted that the FT3 level decreases in HBV patients, while the FT3 and FT4 levels increase in HCV patients (Orságová et al., 2014). More specifically is that along with the increasing inflammatory grade, the level of TT3 primary increased and then decreased, but only the increased level was significantly statistic (Hu et al., 2020). Interestingly, HBV/HCV coinfection elevates the probability of thyroid dysfunction (Ji et al., 2016). Meanwhile, one of the major problems with interferon therapy in hepatitis is the occurrence of aberrant TSH, T3, and T4 values, as well as autoantibodies and thyroid diseases (Ignatova et al., 1998; Orságová et al., 2014; Karwowska et al., 2018). Nevertheless, Huang MJ et al. (1999) indicated that seropositivity of thyroid autoantibodies should not be a contraindication to IFN therapy in HCV-infected patients. Similarly, a recent research reported that the antithyroid antibodies do not cause severe autoimmune disorders in children with chronic HBV infection and merely associated with subclinical hypothyroidism (Kansu et al., 2004).

### Downregulated TH/TR Function Ameliorates Acute Liver Failure

Acute liver failure (ALF), characterized by elevated liver biochemistry, coagulopathy, and hepatic encephalopathy (HE) but with no underlying chronic liver disease (CLF), is a severe and complex clinical syndrome (Lopes and Samant, 2021). The TH/TR axis may be involved in ALF. On the one hand, type A HE is strongly related to low TSH in ALF patients with a concerning poor survival rate (Anastasiou et al., 2015; Wang et al., 2017). HINAT ACLF has proposed liver failure incorporating TSH into the standard (Feng and Shi, 2018; Wu et al., 2018). Interestingly, type C HE often happens to patients with cirrhosis and lower T3 and T4 levels (Wang et al., 2017). In addition, ALF induced by surgical liver devascularization in female pigs observed a decrease in serum-free T3 and T4 as well as TRα protein levels (Kostopanagiotou et al., 2009). Intriguingly, thioacetamide-induced ALF promotes hepatocyte proliferation in response to T3 in the rat (Malik et al., 2006). On the other hand, hypothyroidism prevents immune-mediated acute liver injury in mice, subsequently elevating TSH levels and survival rates and declining serum liver enzymes, blood ammonia, and prothrombin time. It has been reported that a patient with ALF results from non-controlled hyperthyroidism (Sousa Domínguez, 2015). Clinically, plasma exchange is an effective method to eliminate TH in acute liver failure with thyroid storm (Zeng et al., 2017). Mechanistically, low T3 and T4 levels in hypometabolism-associated hypothyroidism link to inflammation and oxidative stress (Bruck et al., 1998). In general, high TSH levels and low TH/TR functions manifest a protector in ALF.

### TH/TR Axis Improves Liver Fibrosis

Liver fibrosis is characterized by chronic inflammation and fibrous scar formation in the liver, finally resulting in hepatocyte deficiency and loss of hepatic function (Erhardtsen et al., 2021). Advanced fibrosis is associated with decreased serum FT3 levels (Du et al., 2021). As an independent risk factor, an elevated TSH level is significantly correlated with the risk of fibrosis (Martínez-Escudé et al., 2021). In a recent study, compared with 12.19% in chronic hepatitis C (CHC) patients without thyroid disease (TD), severe fibrosis is found at 92.85% among CHC patients with TD (Biciusca et al., 2020). However, studies have suggested that hypothyroidism was not highly associated with fibrosis (D'Ambrosio et al., 2021). Treating with TRβ agonist resmetirom in advanced NASH with fibrosis mice have lower α-smooth muscle actin, fibrogenesis-involved genes, and markers of fibrosis, especially including liver stiffness and N-terminal type III collagen pro-peptide (PRO-C3), which indicate that resmetirom can improve fibrosis (Harrison et al., 2021; Kannt et al., 2021). As a result, the TH/TR axis ameliorates liver fibrosis, although the deeper connection between hepatic fibrosis and the TH/TR axis needs more exploration.

### TH May Accelerate Alcoholic Liver Disease

Alcoholic liver disease is caused by long-term heavy drinking, initially manifesting as fatty liver, and hepatocyte necrosis, then developing into alcoholic hepatitis, liver fibrosis, cirrhosis, and liver failure (Wang and Mu, 2021). Papineni et al. (2017) indicated that TH-free T3 (fT3) decreases in alcoholic hepatitis and cirrhosis while fT3 and fT4 increase in chronic alcoholic liver disease patients after treatment. More specifically speaking, low fT3 not only probably reflects the severity of liver disease, the degree of liver damage but may also increase the withdrawal effects and craving for alcohol (Nomura et al., 1975; Israel et al., 1979; Burra et al., 1992). However, it has been reported that alcohol and TH also might cause a hypermetabolic state of the liver and liver cell damage. Accordingly, antithyroid drugs can cure alcoholic fatty liver *via* inhibiting ethanol metabolic rate (EMR) in chronic ethanol-consuming patients (Szilagyi et al., 1983). Overall, the TH/TR axis may aggravate alcoholic liver disease.

### TH/TR Axis Ameliorates Liver Injury

Liver injury is caused by multiple factors mainly including some drugs, poisons, or chronic liver, and extrahepatic diseases (Yamamoto, 1995). In CCl4-induced liver injury in rats, the serum T3 level is reduced due to the decreased release of T3 from liver cells rather than a decreased conversion of T4 to T3 (Ikeda et al., 1986). In general, the TH/TR axis protects against liver damage, and thyroid disorder aggravates the development of liver injury. From the protective aspect, T3 replenishment protects against liver injury *via* improving oxidative stress, cell ferroptosis, detoxification, and increasing drug transport proteins expression, inflammatory factors, autophagy, and lipid metabolism. DNA damage generated by reactive oxygen species upregulates 8-hydroxy-2-deoxyyguanosine (8-OHdG) levels. During diabetes, hypothyroidism, and hypothyroidism with diabetes, the use of TH downregulates the level of 8-OHdG, protein carbonyl content (PCO), protein oxidation, and advanced oxidation protein products (AOPPs) (Altan et al., 2010). In addition, TH synergizes with methylprednisolone (MP) to improve oxidative stress and liver damage and then realizing anti-inflammatory and antioxidant effects (D’Espessailles et al., 2013). Intriguingly, T3 scavenges lipid peroxyl free radicals and improves cell ferroptosis in the LPS/galactosamine-induced liver injury mouse model (Mishima et al., 2020). Moreover, the combined supplementation of T3 and n-3 polyunsaturated fatty acid (n-3 PUFA) in rat decreases ischemia-reperfusion (IR) liver injury and oxidative stress. From the aggravating aspect, hyperthyroidism promotes liver injury. Otherwise, thionamides, methimazole, and propylthiouracil are associated with drug-induced liver injury (LiverTox, 2012; Yan et al., 2017) (Table 2). In a word, the activation of the TH/TR axis can ameliorate liver injury.

**TABLE 2:** Regulatory mechanism of the TH/TR axis alleviates acute liver injury. The regulatory mechanisms are clarified into three categories, including the effects of protection or exacerbation, treating factors, and changed factors. TH downregulates 8-OHdG, PCO, and AOPP levels. TH can also synergize with MP to improve oxidative stress and liver damage and realize anti-inflammatory and antioxidant effects. T3 scavenges lipid peroxyl free radicals and improves cell. The combined supplementation of T3 and n-3 PUFA was given to rats to decrease IR liver injury and oxidative stress. T3 treatment recovers NF-κB activity, STAT3, TNF-α, and haptoglobin and increases liver GSH depletion and protein oxidation protection against IR. T3 upregulates the liver redox-sensitive nuclear transcription factor Nrf2 DNA, detoxification, and drug transport proteins expression, especially including protein levels of Eh1, NQO1, GST Ya, GST Yp, MRP-2, MRP-3, and MRP-4. The inactivation of Kupffer cell by GdCl3 can suppress T3-induced oxidative stress, thus ameliorating the development of liver injury. T3 induces liver PC against IR supported by triggering AMPK, ultimately accelerating the depletion of inflammatory factors such as hepatic NLRP3 and IL-1β. T3 induces hepatocyte proliferation in toxic liver injury. T3 injection protects liver IR damage by enhancing MEK/ERK/mTORC1 mediated autophagy. TH-induced MAO inhibitors inhibit the activity of MAO protecting against liver injury. The accumulation of TR mRNA may remove negative influences in fluoride-related liver injury *via* preventing disruption of lipid metabolism, oxidative damage, and apoptosis. Hyperthyroidism promotes liver injury. Thionamides, methimazole, and propylthiouracil are associated with drug-induced liver injury. The Yinning Tablet restores the expression of antiapoptotic Bcl-2 cytosol cytochrome c protein overexpression and downregulates the expression of l-thyroxine-induced overexpressed caspase-9, -8, -3, proapoptotic BAX and Dio1, thus ameliorating TH-induced liver injury in rats through regulating mitochondria-mediated apoptotic signals. (The arrows indicate factors are unregulated or downregulated.)

Effects of protection	Treating factor	Changed factor
Oxidative stress	T3 + insulin GdCl3	8-OHdG, PCO, and AOPPs ↓
		Inactivation of Kupffer cells
Cell ferroptosis	T3	Lipid peroxyl free radicals ↓
Detoxification	T3	Nrf2, Eh1, NQO1, GST Ya, and GST Yp
		MRP-2, -3, -4 ↑
Inflammation	TH + MP	—
Autophagy	T3	MEK/ERK/mTORC1 ↑
Lipid metabolism apoptosis	Yinning Tablet	TR ↑
		TR ↑
		Bcl-2 and cytochrome c protein ↑
		caspase-9, -8, -3, proapoptotic BAX, and Dio1 ↓
DNA and protein damage	T3 + insulin	—
IR	TH + nPUFA	NF-kB, STAT3, THF-α, and haptoglobin ↓
	—	GSH depletion and protein oxidation ↑
	T3	AMPK ↑
		NLRP3 and IL- 1β ↓
Other mechanisms	T3	MAO ↓

## The Regulatory Mechanisms Underlying TH/TR Axis May Supply Novel Treatment Methods for Liver Diseases

The regulatory mechanism of the TH/TR axis in liver diseases has been gradually elucidated. However, the understanding of the regulatory mechanism of the TH/TR axis in liver diseases is not entirely clear, which is still being explored. The study on the regulatory mechanism of the TH/TR axis in hepatic diseases is helpful to reveal the importance in liver diseases.

### MAFLD

In MAFLD, the deiodinase family members, especially including types 1, 2, and 3 iodothyronine deiodinases (Dio1, 2, and 3) and responsible for the activation and inactivation of TH, can modulate the TH/TR axis. As hepatic enzymes, Dio1 and Dio2 convert T4 to T3 and increase T3/T4 levels. Conversely, Dio3 inactivates TH. To be specific, the activation of Dio3 activates hypoxia-inducible factor 1α (HIF-1α), thus inhibiting T3 signaling and the metabolic rate (Bianco and da Conceição, 2018; Luongo et al., 2019; Russo et al., 2021). Increased Dio1 level promotes β-oxidation of fatty acid and oxidative phosphorylation, then preventing hepatocyte steatosis. Moreover, the level of Dio1 mRNA depends on the dietary conditions. When fed a normal chow diet (NCD), Leprdb mice grows up with severe steatosis with only mild inflammation. The depletion of Dio1 in Leprdb mice upregulates hepatic Tnfa and Co1a1 mRNA levels, which are inflammation and fibrosis biomarkers, respectively (Bruinstroop et al., 2021). Genes related to reverse cholesterol transport and lipase activity decrease with the downregulation of Dio2 in rats (Russo et al., 2021). The sites of *de novo* DNA hypermethylation (H sites) disrupt long-distant chromatin interactions, looping enhancers, and promoters in hepatocytes. TH produced from Dio2 activation depletes H3K9me3 and interferes with the formation of more than a thousand H sites, subsequently maintaining the liver development and function (Fonseca et al., 2021). In general, the TH/TR axis modulated by the deiodinases may delay MAFLD progression. Nevertheless, the regulatory mechanism of the TH/TR axis in MAFLD remains to be further studied (Figure 1).

### HCC

The TH/TR axis modulates cyclin-dependent kinase (CDK) and cyclins, MicroRNAs (miRNAs), long non-coding RNA (lncRNA), TGF-β signaling, hedgehog (Hh) (relying on the local deiodinase expression), and other tumor-related genes and -proteins to be involved in the growth, proliferation, and metastasis of HCC (Manka et al., 2018).

In addition, CDKs and regulatory subunits cyclins regulate the cell cycle in mammalian. P21, as a CDK inhibitor, halts G1/S and G2/M transitions of cell cycle progression by inhibiting CDK4,6/cyclin-D and CDK2/cyclin-E, respectively (Karimian et al., 2016). The inhibition of HCC cells growth and proliferation is dependent upon the activation of P21 by the TH/TR axis. A recent study reported that the activation of the TH/TR axis upregulates endoglin in HCC cells, thus restraining P21 polyubiquitination-induced cell proliferation (Lin et al., 2013a). In addition, the TH/TR axis inhibits hepatoma cell growth *via* repressing UHRF1 and relieves UHRF1-mediated P21 silence (Wu et al., 2015). Furthermore, TH induces the miR-214-3p expression, followed by interfering with the proto-oncogene serine/threonine-protein kinase (PIM-1) and activating P21, thus blocking cell proliferation (Huang et al., 2017). Other CDKs and cyclins also involve in the regulatory mechanism in HCC in a TH/TR axis-dependent fashion. Ezequiel et al. (Ridruejo et al., 2021) suggested that hexachlorobenzene (HCB) is an endocrine disruptor and a liver tumor promoter. In the HepG2 cell line, the depletion of HCB by TH leads to the downregulation of the TGF-β1/pSMAD-2/3 signaling pathway, thus increasing Dio1 levels and decreasing p21 and P27, ultimately suppressing cell proliferation. Beyond that, the silence of TGF-β mice promote the proliferation by increasing the expressions of CDK2, cyclin E, and cyclin A, as well as decreasing the expression of CDKn1a/p21 (Baek et al., 2010). It has been reported that depleting forkhead box M1 (FOXM1) by TH interferes with oncogenic expression of cyclin D1, cyclin E, and CDK2, thereby inhibits HCC cells proliferation (Barrera-Hernandez et al., 1999; Wu et al., 2020). These pathways all manifest that the TH/TR axis closely interacts with p21, CDKs, and its regulatory subunits cyclins to affect cell proliferation in HCC.

Moreover, miRNAs, a class of evolutionarily conserved non-protein-coding small RNA, are responsible for regulating gene expression at the translation level (Oura et al., 2020). The TH/TR axis regulates miRNAs, thereby producing various effects in HCC. For example, the downregulation of the TH/TR axis induces nodule regression and the increased expression of targeted microRNA, miR-27a, miR-181a, miR-204a, and miR-181a in the resistance-hepatocyte rat model (R-H model) and human cirrhotic peritumoral tissue (Frau et al., 2015). Beyond that, miR-214-3p, miR-130b, miR-17, miR-21, miR-424, and miR-503 also participate in the regulation of the TH/TR axis-mediated liver cancer (Huang Y. H. et al., 2013; Lin et al., 2013b; Ruiz-Llorente et al., 2014; Lin et al., 2015). Thus, miRNAs have the potential to be targets in TH/TR-involved HCC.

Furthermore, lncRNA, the human major transcriptional genome, is a length greater than 200 nucleotides, which is a non-coding protein (Dang et al., 2015). A recent study has reported that compared to the non-tumor samples, the expression of lncRNA related genes including MSC-AS1, POLR2J4, EIF3J-AS1, SERHL, RMST, and PVT1 are upregulated in tumor samples. Beyond that, lncRNA genes mostly cluster in the TGF-β signaling pathway, internal ribosome entry pathway, granzyme A mediated apoptosis, FAS signaling pathway, calcium signaling by HBx, and p38/MAPK signaling pathway (Gu et al., 2019). It has been reported that lncRNA CRNDE and lncRNA SNHG7 are independent risk factors of synchronous colorectal liver metastasis (SCLM), which also predict a high tumor recurrence rate (Zhang P. et al., 2020). Since lncRNA is associated with the occurrence of tumor, it is plausible that lncRNA may intimately be regulated by the TH/TR axis. Indeed, the TH/TR axis is related to lncRNA in HCC. For instance, brain cytoplasmic RNA 1 (BCYRN1 or BC200) is widely expressed in tumors. BC200 is also inhibited by T3/TR, then downregulating the expressions of CDK2, cyclin E1, and cyclin E2 and upregulating P21, thereby repressing cell growth and tumor sphere formation and preventing the evolvement of HCC (Lin et al., 2018). Otherwise, the downregulation of taurine upregulated gene 1 (TUG1) by the TH/TR axis also cause AFP mRNA, cyclin E, and H3K27me3 silence and cell growth inhibition (Lin et al., 2020b).

Finally, other TH/TR-related genes and -proteins are involved in HCC. Thyroid hormone receptor-interacting proteins (TRIP), the Zyxin family of LIM proteins, is responsible for regulating transcription of TR. Significantly, the transcriptional activation of the TH/TR axis in HCC may depend on TRIP. Specifically, when TRIP6 activates FOXC1, migration, invasion, and proliferation are strongly promoted. It is also found that TRIP6 induces cyclin D1 expression, decreases p21 and p27 activation, and HCC cell proliferation arrest (Lee et al., 1995; Zhao et al., 2017; Wang et al., 2020). Moreover, the downregulation of TRIP13 impairs the NHEJ repair process, increased apoptosis, and cell cycle arrest at the S-phase, ultimately inhibiting the proliferation, migration, and invasion of HCC cells (Ju et al., 2018). In addition, pituitary tumor transforming gene 1 (PTTG1) is silenced by Sp2, which is negatively mediated by T3/TR in Hep3B hepatoma cells (Chen et al., 2008). Ndrg2 is a Myc suppressor gene. The activation of V-erbA leads to the depletion of Ndrg2, thus exacerbating tumor invasion and metastasis (Ventura-Holman et al., 2011). As a tumor-associated protein, lipocalin 2 (Lcn2) can activate the Met/FAK pathway in a TH/TR axis-dependent manner, thus enhancing tumor cell migration and invasion (Chung et al., 2015). Intriguingly, T3/TR/MEK/ERK/NUPR1/PDGFA cascade may play a vital role in hepatocarcinogenesis. Consistently, T3/TR positively regulates nuclear protein 1(NUPR1) *via* binding to the NUPR1 promoter regions, therefore promoting vascular invasion (Chen et al., 2019). Recently, it has been found that increasing thyroid hormone responsive (THRSP) prevents the silence of the ERK/ZEB1 signaling pathway and inhibits the process of epithelial-to-mesenchymal transition, subsequently preventing hepatocellular carcinogenesis (Hu et al., 2021). A secreted protein named Dickkopf 4 (DKK 4) antagonizes the Wnt signal pathway and inhibits tumor metastasis, which is dependent upon the activation of the T3/TR axis (Chi et al., 2013).

**FIGURE 2:**
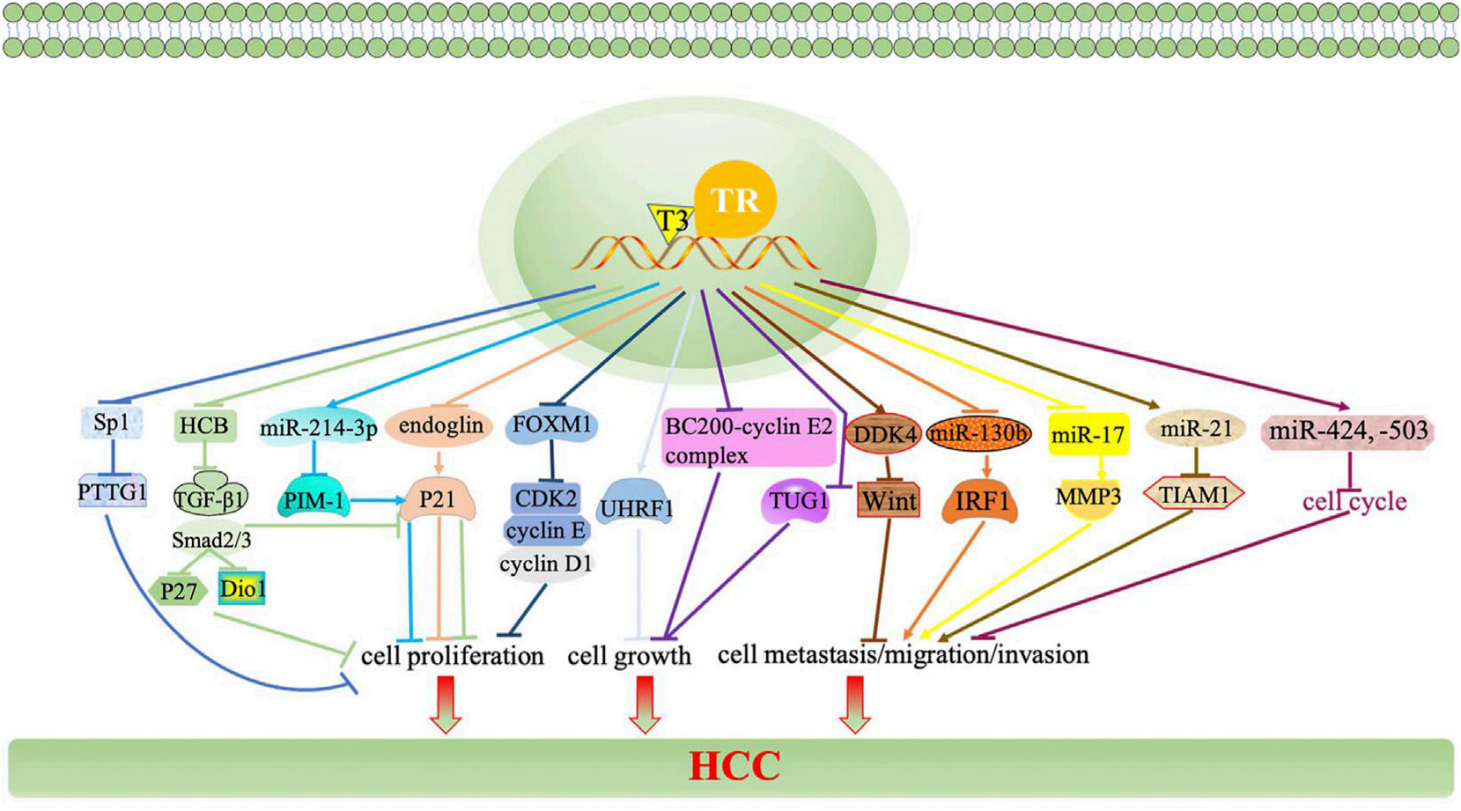
TH/TR axis is involved in HCC growth, proliferation, invasion, and metastasis. The TH/TR axis upregulates endoglin, thus restraining P21 polyubiquitination-induced cell proliferation. The TH/TR axis inhibits hepatoma cell growth *via* repressing UHRF1 and relieves UHRF1-mediated P21 silence. TH induces the miR-214-3p expression, followed by interfering with PIM-1 and activating P21, thus blocking cell proliferation. The depletion of HCB by TH downregulates the TGF-β1/pSMAD-2/3 signaling pathway, thus increasing Dio1 levels and decreasing p21 and P27, ultimately suppressing cell proliferation. The silence of TGF-β mice promote the proliferation by increasing the expressions of CDK2, cyclin E, and cyclin A, as well as decreasing the expression of CDKn1a/p21. Depleting FOXM1 by TH interferes with cyclin D1, cyclin E, and CDK2, thereby inhibiting HCC cell proliferation.

**FIGURE 3:**
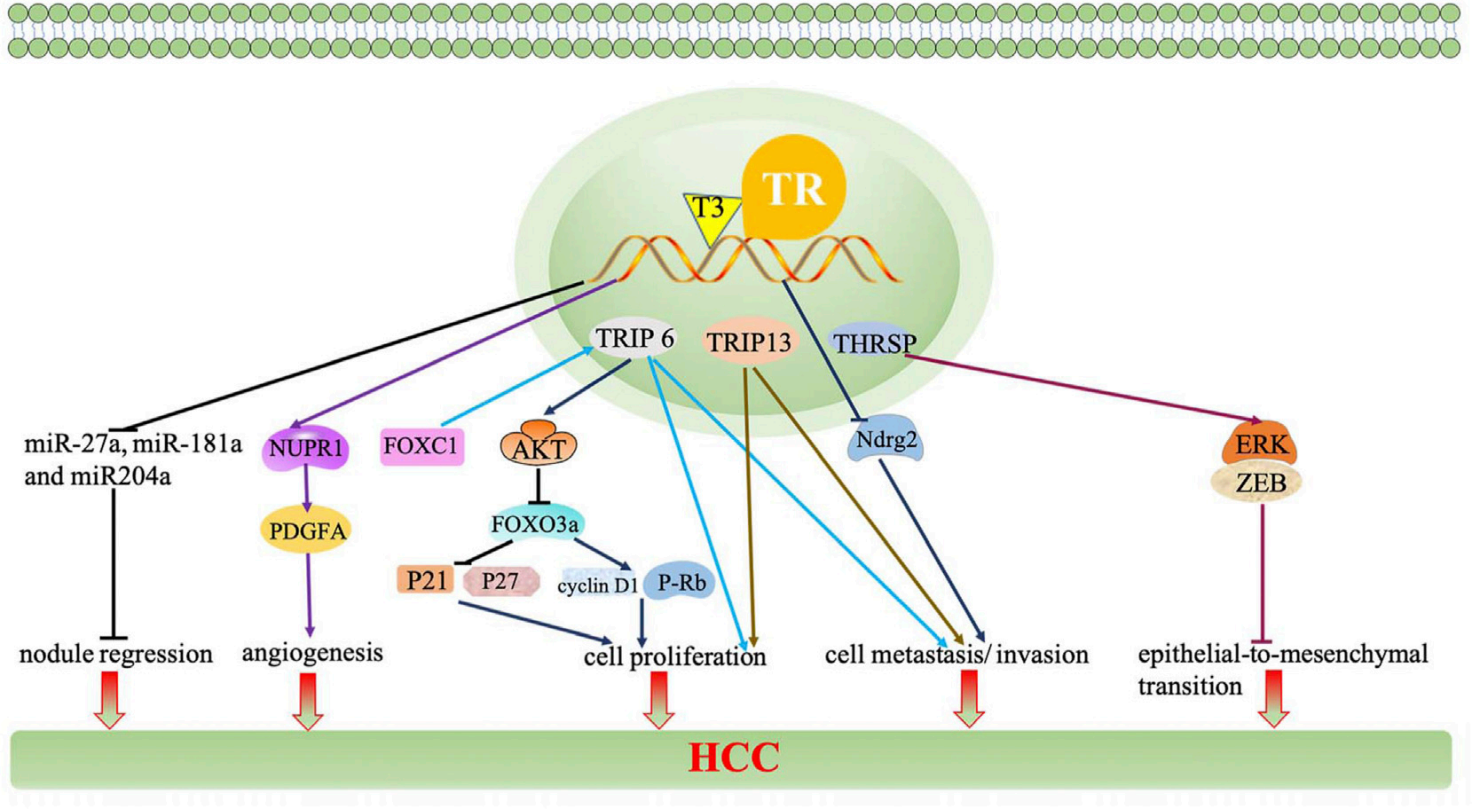
The TH/TR axis inhibits nodule regression and decreases the expressions of miR-27a, miR-181a, and miR-204a. BC200 is inhibited by T3/TR and downregulates the expressions of CDK2, cyclin E1, and cyclin E2 and upregulates P21, thereby repressing cell growth. The downregulation of TUG1 by the TH/TR axis causes AFP mRNA, cyclin E, and H3K27me3 silence and cell growth inhibition. When TRIP is activated by FOXC1, migration, invasion, and proliferation are strongly promoted. TRIP6 induces the AKT signaling pathway, thereby preventing FOXO3a overexpression-induced cyclin D1 interference, p21 and p27 activation, and HCC cell proliferation arrest. The downregulation of PTTG1 is silenced by Sp2, which is negatively mediated by T3/TR. The activation of V-erbA leads to the depletion of Ndrg2, thus exacerbating tumor invasion and metastasis. Lcn2 can activate the Met/FAK pathway in a TH/TR axis-dependent manner, thus enhancing cell migration and invasion. T3/TR/MEK/ERK/NUPR1/PDGFA cascade may play a vital role in hepatocarcinogenesis. T3/TR upregulates NUPR1 *via* binding to the NUPR1 promoter regions, therefore promoting vascular invasion. THRSP prevents silence of the ERK/ZEB1 signaling pathway and inhibits the process of epithelial-to-mesenchymal transition. DKK 4 antagonizes the Wnt signal pathway and inhibits tumor metastasis, which depends upon the activation of the T3/TR axis.

In short, the TH/TR axis have paradoxical role in the growth, proliferation, invasion, metastasis, and migration of HCC. A relevant regulatory mechanism of the TH/TR axis in HCC remains to be further explored (Figures 2, 3).

### Liver Injury

In liver injury, the studies show that T3 treatment recovers NF-κB activity, signal transducer, and activator of transcription 3 (STAT3), TNF-α and haptoglobin and increases liver GSH depletion and protein oxidation protecting against IR (Fernández et al., 2007; Mardones et al., 2012). A study exhibited that T3 upregulates the liver redox-sensitive nuclear transcription factor erythroid 2-related factor 2 (Nrf2) DNA, detoxification, and drug transport proteins expression, mainly including protein levels of epoxide hydrolase 1 (Eh1), NADPH-quinone oxidoreductase 1 (NQO1), glutathione-S-transferases Ya (GST Ya), GST Yp, multidrug resistance-associated proteins 2 (MRP-2), mrp-3 ,and MRP-4 in male Sprague–Dawley rats, which may indicate the hepatocyte protective mechanism in liver injury attributed to ROS and chemical toxicity (Cornejo et al., 2013). In addition, the inactivation of Kupffer cell by gadolinium chloride (GdCl3) can suppress T3-induced oxidative stress, thus ameliorating the development of liver injury characterized by neutrophil infiltration and necrosis (Simon-Giavarotti et al., 2002). Accumulating evidence has demonstrated that T3 induces liver preconditioning (PC) against IR supported by triggering AMP-activated protein kinase (AMPK), ultimately accelerating the depletion of inflammatory factors such as hepatic NLRP3 and IL-1β (Fernández et al., 2009; Vargas and Videla, 2017). T3 induces hepatocyte proliferation in toxic liver injury (Malik et al., 2006). T3 injection protects liver IR damage by enhancing MEK/ERK/mTORC1-mediated autophagy in male C57BL/6 mice (Yang et al., 2015). In addition, TH-induced monoamine oxidase (MAO) inhibitors inhibit the activity of MAO protecting against liver injury in rats (Obata and Aomine, 2009). In addition to the TH/TR axis also prevents liver damage. It has been discovered that the accumulation of TR mRNA may remove negative influences in fluoride-related liver injury *via* preventing disruption of lipid metabolism, oxidative damage, and apoptosis (Bo et al., 2018). Particularly, traditional Chinese medicine is also involved in improving the TH/TR axis-induced liver injury. To be specific, the Yinning Tablet restores the expression of antiapoptotic Bcl-2 cytosol cytochrome c protein and downregulates the expression of l-thyroxine-induced overexpressed caspase-9, -8, -3, proapoptotic BAX and Dio1, thus ameliorating TH-induced liver injury in rats *via* regulating mitochondria-mediated apoptotic signals (Yang et al., 2020).

### Other Liver Diseases

The TH/TR axis may be correlated with hepatitis. Some studies have demonstrated that ubiquitin-specific protease 18 (USP18), known as UBP43, participates in gene regulations of the TH signaling pathway. Li et al, (2017) discovered that USP18 regulates the signaling of antivirus by the TH signaling pathway, prolactin signaling pathway, insulin resistance and complement, and have crosstalk among them. Also, the thyroid hormone-uncoupling protein (TRUP) gene and thyroid hormone receptor-associated protein 150 alpha gene are associated to the integration of HBV DNA into liver cell DNA, which are the key regulators of cell proliferation and viability (Gozuacik et al., 2001; Paterlini-Bréchot et al., 2003). In recent years, the relationship between the TH/TR axis and hepatitis of HBV and HCV infection has been gradually elucidated. However, understanding of the antiviral mechanism of the TH/TR axis is not entirely clear, which is still being explored.

**FIGURE 4:**
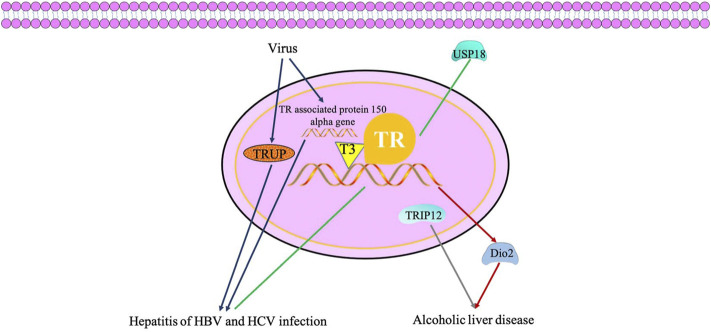
TH/TR axis may be involved in hepatitis of hepatitis B virus and hepatitis C virus infection and accelerates alcoholic liver disease. USP18 regulates the signaling of antivirus by the thyroid hormone signaling pathway. TRUP gene and thyroid hormone receptor associated protein 150 alpha gene are associated to the integration of HBV DNA into the liver cell DNA, which are key regulators of cell proliferation and viability. TH has been proven to increase the level of Dio2, thereby elevating susceptibility to hepatic steatosis in a model of alcoholism. The mRNA level of TRIP12 is significantly different in alcohol liver disease.

Furthermore, the TH/TR axis is associated with alcohol-related hepatic alterations. For example, TH has been proven to increase the level of Dio2, thereby elevating susceptibility to hepatic steatosis in a model of alcoholism (Fonseca et al., 2019; Hernandez, 2019). In addition, the mRNA level of TRIP12 is significantly different in alcohol-feed (AF) and control pair-feed (PF) mice (Zhang et al., 2018) (Figure 4).

## Conclusion

Here, we amply reviewed the function of the TH/TR axis in hepatic diseases. The TH/TR axis may protect against metabolic-associated fatty liver disease, hepatitis B virus and hepatitis C virus infection, liver fibrosis, and drug-induced and extrahepatic liver injury but may accelerate the development of acute liver failure and alcoholic liver disease. Meanwhile, the axis has a dual role in hepatocellular carcinoma. As a result, targeting the TH/TR axis should be considered for treating liver disease, which may be a promising disease-reversing strategy for patients.

## Prospections

The total T3 in different stages of inflammation exhibits a trend of first increasing and then decreasing (Hu et al., 2020). Although there is no statistical significance of the total T3 decreases, it is still trustworthy, owing to the fact that the initial stage of TH also occurs booming in acute liver failure. Subsequently, the later declination may be due to the adjustment and balance of the body in different periods. In this retrospective study, the patient’s aggravation is not serious, and the samples are not large enough (*n* = 6), thus leading to an insignificant decrease.

TH protects liver cells from damage by mediating the HBV/HCV infectious signaling pathway and then improves hepatitis-related carcinogenic transformation. TSH and TT3 are promising aspects to be included in the evaluation criteria for inflammatory activities and served as biological markers to reduce the proportion of liver biopsy and the medical burden. Moreover, the treatment of interferon is not strongly related to autoimmune diseases except for thyroid. However, virus infection and antivirus endeavors lead to format a certain proportion of thyroid autoantibodies and related thyroid diseases such as hypothyroidism, and this cannot be ignored.

To date, it is reported that the mutation of the TRβ gene in thyroid hormone resistance patients leads to the impairment of TRβ signals in the hepatic steatosis. The mutation type of the THRβ gene is the substitution of glycine by arginine at position 243 (R243Q) of TRβ. Compared with patients with WT relatives, serum T3 and T4 of RTH β patients are higher than the upper limit of a reference range. However, there is no significant difference in TSH. They also found that the liver fat content, serum free fatty acids, and HDL cholesterol were higher (Chaves et al., 2021). At present, the fragments related to lipid metabolism are all located in the hinge region of TR. In the future, gene mutations may be used to discover the region that regulates TR lipid metabolism in the hinge region, as well as other regions and their functions, and this pathway exactly can become the therapy targets of NASH/NAFLD and HCC.

Post-translational modifications participate in the occurrence and development of liver disease. Both TRα and TRβ are regulated by the level of PTM, including SUMOylation (Liu and Brent, 2018). The small ubiquitin-like modifier (SUMO) family, existing widely in eukaryotes, is a highly conserved post-translational modification protein that regulate lipid metabolism, inflammatory response, bile acid homeostasis, autophagy, and other related biological functions in nuclear receptors (Zeng et al., 2020; Liu et al., 2021). The research team found that the sumo-3 protein (mainly expressed in the nucleus) and TR receptor have significantly upregulated in oleic acid (OA)-induced NAFLD in WRL68 cells and human liver tissue models (published in Chinese journals). TR can be SUMOylated (Anyetei-Anum et al., 2019). At the same time, nuclear autophagy can improve metabolic disorders (Fu et al., 2018). TR SUMOylation may associate with nuclear autophagy to improve metabolic disorders and prospectively become a therapeutic target in NAFLD.

In summary, the TH/TR axis provides effective insights into the treatment of hepatic diseases. The research and application prospects for the TH/TR axis in liver diseases are promising. TH/TR may provide new potential therapeutic targets

## Glossary

TH/TR axis: thyroid hormone/thyroid hormone receptor axis
T4: thyroid hormones 3,5,3′,5′- tetraiodothyronine or thyroxine
T3: 3,5,3′-triiodothyronine
AEV: avian erythroblastosis virus
TRE: thyroid hormone-response element
TSH: thyroid-stimulating hormone
PCNA: proliferating cell nuclear antigen
cdk2: cyclin-dependent kinase 2
PARP: Poly (ADP-ribose) polymerase
PH: partial hepatectomy
MAFLD: metabolic-associated fatty liver disease
HCC: hepatocellular carcinoma
HBV: hepatitis B virus
HCV: hepatitis C virus
ALF: acute liver failure
GC-1: sobetisome
Eprotirome: KB2115
MetS: metabolic syndrome
TPOAb: thyroid peroxidase antibody
SBP: systolic blood pressure
DBP: diastolic blood pressure
FBG: fasting blood glucose
TG: triglyceride
HDLC: high-density lipoprotein
ALT: alanine aminotransferase
AST: aspartate aminotransferase
BUN: urea nitrogen
CR: creatinine
Dio: iodothyronine deiodinases
HIF-1α: hypoxia-inducible factor 1α
NCD: normal chow diet
H sites: sites of *de novo* DNA hypermethylation
CDK: cyclin-dependent kinase
miRNAs: microRNAs
lncRNA: long non-coding RNA
Hh: Hedgehog
PIM-1: the proto-oncogene serine/threonine-protein kinase-1
HCB: hexachlorobenzene
FOXM1: forkhead box M1
R-H model: resistance-hepatocyte rat model
SCLM: synchronous colorectal liver metastasis
BC200: brain cytoplasmic RNA 1
TUG1: taurine upregulated gene 1
TRIP: Thyroid hormone receptor-interacting proteins
PTTG1: pituitary tumor-transforming gene 1
Lcn2: lipocalin 2
NUPR1: nuclear protein 1
THRSP: thyroid hormone responsive
DKK 4: dickkopf 4
USP18: ubiquitin-specific protease 18
TRUP: thyroid hormone uncoupling protein
ALF: acute liver failure
HE: hepatic encephalopathy
CLF: chronic liver disease
CHC: chronic hepatitis C
TD: thyroid disease
PRO-C3: N-terminal type III collagen pro-peptide
PBC: primary biliary cirrhosis
fT3: TH-free T3
EMR: ethanol metabolic rate
AF: alcohol-feed
PF: pair-feed
8-OHdG: 8-hydroxy-2-deoxyyguanosine
PCO: protein carbonyl content
AOPPs: advanced oxidation protein products
MP: methylprednisolone
n-3 PUFA: n-3 polyunsaturated fatty acid
IR: ischemia-reperfusion
STAT3: signal transducer and activator of transcription 3
Nrf2: nuclear transcription factor erythroid 2-related factor 2
Eh1: epoxide hydrolase 1
NQO1: NADPH-quinone oxidoreductase 1
GST: glutathione-S-transferases
MRP: multidrug resistance-associated proteins
GdCl3: gadolinium chloride
PC: preconditioning
AMPK: AMP-activated protein kinase
MAO: monoamine oxidase
R243Q: substitution of glycine by arginine at position 243
SUMO: small ubiquitin-like modifier
OA: oleic acid
